# Intranasal Application of *Lactococcus lactis W136* Is Safe in Chronic Rhinosinusitis Patients With Previous Sinus Surgery

**DOI:** 10.3389/fcimb.2020.00440

**Published:** 2020-10-12

**Authors:** Leandra Mfuna Endam, Saud Alromaih, Emmanuel Gonzalez, Joaquin Madrenas, Benoit Cousineau, Axel E. Renteria, Martin Desrosiers

**Affiliations:** ^1^Centre de Recherche du Centre Hospitalier de l'Université de Montréal (CRCHUM), Montreal, QC, Canada; ^2^Faculty of Medicine, King Saud University, Riyadh, Saudi Arabia; ^3^Department of Microbiology and Immunology, Microbiome and Disease Tolerance Centre (MDTC), McGill University, Montreal, QC, Canada; ^4^The Lundquist Institute for Biomedical Innovation at Harbor-UCLA Medical Center, Torrance, CA, United States; ^5^Division of Otolaryngology-Head and Neck Surgery, Centre Hospitalier de l'Université de Montréal (CHUM), Montreal, QC, Canada; ^6^Los Angeles Biomedical Research Institute at Harbor-UCLA Medical Center, Torrance, CA, United States

**Keywords:** chronic rhinosinusitis (CRS), probiotics, *Lactococcus lactis W136*, sinus irrigation, CRS treatment, sinus microbiome, refractory CRS

## Abstract

**Objective:** Modulation of the dysbiotic gut microbiome with “healthy” bacteria via a stool transplant or supplementation is increasingly practiced, however this approach has not been explored in the nasal passages. We wished to verify whether *Lactococcus lactis W136* (*L. lactis W136)* bacteria could be safely applied via irrigation to the nasal and sinus passages in individuals with chronic rhinosinusitis (CRS) with previous undergone endoscopic sinus surgery, and whether this was accompanied by bacterial community flora modification.

**Study Design:** Prospective open-label pilot trial of safety and feasibility.

**Setting:** Academic tertiary hospital center.

**Subjects and Methods:** Twenty-four patients with CRS refractory to previous medical and surgical therapy received a 14-day course of BID sinus irrigations containing 1.2 × 10^9^ CFU live *L. lactis W136*. Patients were monitored for safety using questionnaire, sinus endoscopy, otoscopy, UPSIT-40 smell testing, and endoscopically-obtained conventional sinus culture and a swab for 16S microbiome profiling.

**Results:** All 24 patients receiving at least one treatment successfully completed treatment. *L. lactis W136* probiotic treatment was safe, with no major adverse events or new infections. Treatment was associated with improvement in sinus symptoms, QOL, and mucosal scores, which remained improved during the subsequent 14-day observation period. Microbiome changes associated with treatment were limited to an increase of the pathobiont *Dolosigranulum pigrum*, a bacteria identified as potentially beneficial in the upper airways. Subgroup analysis suggested differences in microbiomes and responses for CRSsNP and CRSwNP phenotypes, but these did not attain significance.

**Conclusion:** Intranasal irrigation of live *L. lactis W136* bacteria to patients with refractory chronic rhinosinusitis was safe, and was associated with effects on symptoms, mucosal aspect and microbiome composition. Intranasal bacteria may thus find a role as a treatment strategy for CRS.

**Clinical Trials Registration:** www.ClinicalTrials.gov. identifier: NCT04048174.

## Introduction

Chronic rhinosinusitis (CRS) is considered a complex disease, where multiple factors, including inflammatory cell infiltrate, microbiome dysbiosis, and dysfunction of the sinus epithelium interact to initiate and maintain the clinical phenotypes of chronic rhinosinusitis with nasal polyposis (CRSwNP) and chronic rhinosinusitis without nasal polyps (CRSsNP) (Meltzer et al., 2004; Van Zele et al., 2006; Nader et al., 2010; Stephenson et al., 2010). Current treatment options center on the combinations of topical and oral corticosteroids and surgery (Desrosiers et al., 2011). However, even following surgery, endoscopic signs of recurrence are seen in over 50% of the patients by 4 months after surgery, and even post-operative therapy with topical corticosteroid drops or sprays does not prevent the recurrence of disease (Meltzer et al., 2004; Stjarne et al., 2009). These patients, with CRS refractory to medical and surgical therapy, undergo considerable suffering and discomfort and represent a considerable burden to the health care system. Novel alternate therapies are thus urgently required.

The microbiome dysbiosis present in CRS may represent a novel treatment opportunity via microbiome supplementation (Wagner Mackenzie et al., 2017; Chalermwatanachai et al., 2018). Microbiome-based therapies are increasingly common in the digestive tract. Stool “transplants,” where healthy flora from normal donors are introduced into diseased colon, has been shown to control colonic inflammation of various etiologies (Snelling, 2005; Wolvers et al., 2010; Aroniadis and Brandt, 2013). Supplementation with orally administered probiotics for restoring gastrointestinal microbiome has shown varying results in the literature going from little improvement (Kristensen et al., 2016) to being beneficial (Oelschlaeger, 2010; Ferrario et al., 2014; Bjerg et al., 2015; Brahe et al., 2015).

In the sinuses, the microbiome is believed to play a beneficial role in health maintenance. Conventional culture techniques have shown healthy sinuses after ESS to be associated with *S. epidermidis* (Al-Shemari et al., 2007), a Gram-positive coccus. The mechanisms by which *S. epidermidis* promote health in the nose and sinuses remain incompletely described but can be extrapolated from other models. In mice, intranasally administered *S. epidermidis* interfered with *S. aureus* colonization via direct interference or modification of the ecological niche (Abreu et al., 2012; Cleland et al., 2014). Other mechanisms besides direct bacterial interference are believed to be responsible for the beneficial effects seen. In atopic dermatitis (AD), a disorder with pathophysiologic and microbiome features similar to CRS, immunomodulatory effects of the bacteria on epithelium of dendritic cells may also be playing important roles. In AD, *S. epidermidis* is required to dampen inflammation following injury, via interaction of lipoteichoic acid (LTA; a TLR2 ligand present in the capsule of *S. epidermidis*) with innate immune receptors (Lai et al., 2009).

However, the therapeutic potential *S. epidermidis* was tempered by safety concerns regarding the risks of disease production when applied directly to the delicate nasal and sinus passages. Despite its strong potential as a therapeutic commensal, *S. epidermidis* can represent a formidable pathogen in certain settings, notably the neonatal intensive care unit and infection of intrvascular foreign bodies (Moles et al., 2020) This was thus of particular concern in CRS patients, where anatomical barriers to the sinuses have been removed at surgery, and motivated us to search for a suitable candidate which might retain some of the desirable properties of *S. epidermidis*, but with lesser safety concerns.

We thus needed to identify a potentially suitable non-pathogenic Gram-positive coccus for intranasal application. *L. lactis* appears to be a reasonable candidate. It has an excellent safety profile in animal experiments and human use, and shares with *S. epidermidis* the following characteristics believed to be beneficial. A Gram-positive cocci, it possesses a surface capsule rich in LTA motifs, and is free of pathogenic genes. Its safety profile is incontrovertible. *L. lactis has* been used in the food industry for over 100 years (Song et al., 2017), conferring it “Generally Recognized as Safe (GRAS)” status in the US, EU, and Canada (Doty et al., 1984; Schwartz et al., 2016) for oral administration. *In vitro* studies have supported the safety and immunomodulatory capacities of *L. lactis* for intranasal use (Schwartz et al., 2016). Primary epithelial cell cultures raised from sinus mucosa showed no evidence of toxicity when exposed to a supernatant of this strain, while peripheral blood monocyte preparations (PBMC) showed IL-10 induction without evidence of toxicity or excessive Th1-type inflammation (Schwartz et al., 2016). Intranasal administration in mice was also reported to be well-tolerated (Oelschlaeger, 2010).

We wished to verify if topical administration of *L. lactis W136* to the nasal and sinus cavities would be safe for patients with chronic rhinosinusitis refractory to medical and surgical treatment. To this end, we performed an unblinded prospective trial to assess the effects of intranasal administration of *L. lactis* in patients with chronic rhinosinusitis.

## Methods

### Patient Selection

**Figure 1:**
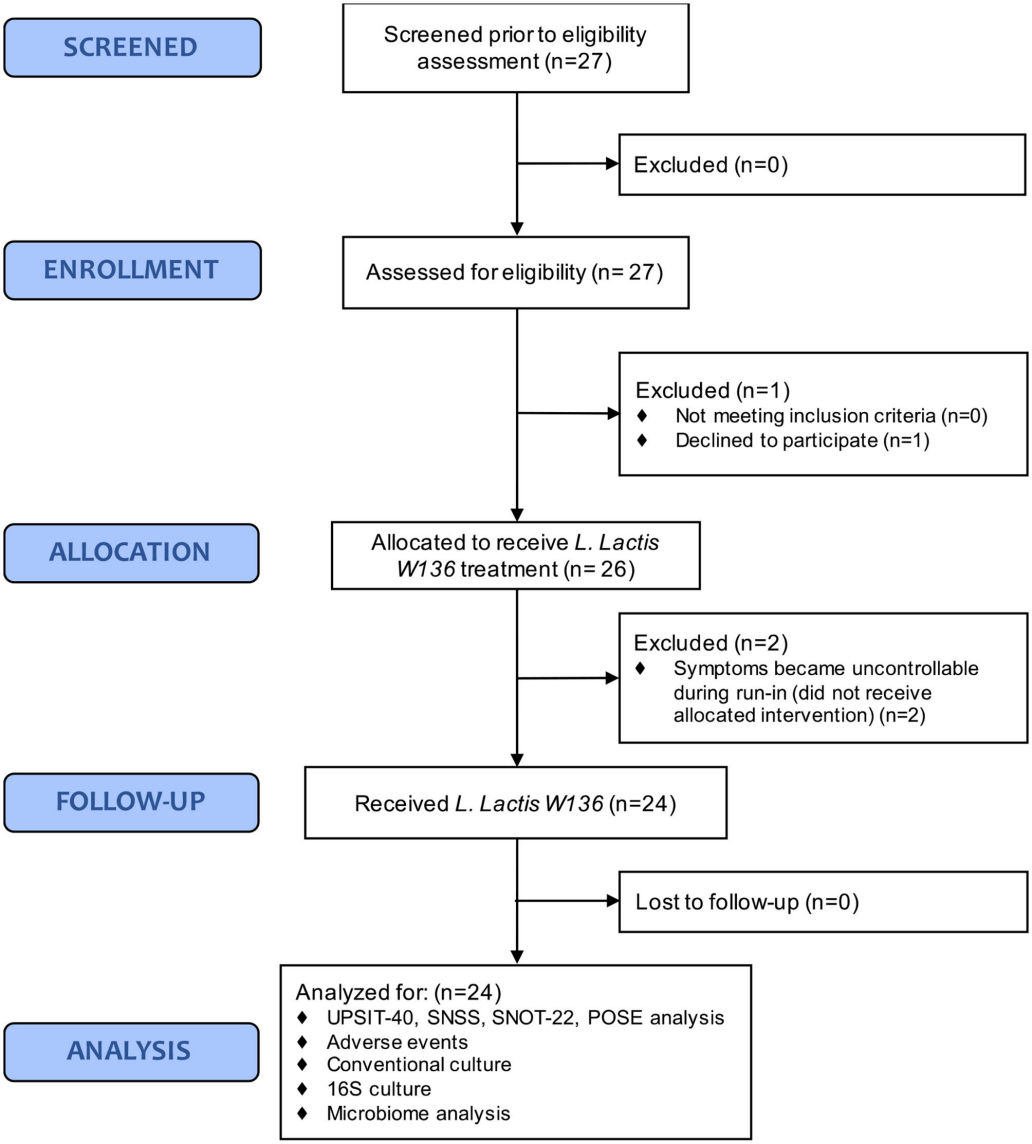
CONSORT statement.

We included patients presenting persistent signs and symptoms of CRSsNP or CRSwNP despite previous technically adequate surgery and continued use of maximal medical therapy, including high-volume budesonide irrigations post-operatively (“refractory” CRS). Excluded were patients <18 years, cystic fibrosis, with technical reasons for ESS failure, active sinus infection with purulence, pain, and/or hyperthermia, or with immune suppression from disease or medication. Complete patient recruitment and enrolment flow chart is presented in Figure 1 as per CONSORT standards.

Sample size needed to detect of side effects occurring at a frequency of 8% or greater with a 95% CI was calculated to be 24 (using ± 1 SD as an estimate of variability). As our primary concern was safety, a number was used which balanced resources available with capacity of identifying frequent side effects.

### Study Design

The trial took place from November 18, 2013 to December 12, 2015 at the Centre Hospitalier de l'Université de Montréal (CHUM), an academic reference center, and was performed by a single ENT surgeon (MD) (Clinicaltrials.gov identifier: NCT04048174.) Patients with CRS refractory to medical and surgical therapy (Meltzer et al., 2004; Desrosiers et al., 2011) were enrolled in a prospective, single-arm, open-label trial. The single arm trial design was selected to ensure maximal patient exposure to *L. lactis W136* and minimize “carry-over” effect from bacterial treatment. While no placebo control was used in this study, all patients underwent a run-in period during which all medications were ceased, including corticosteroid sprays, high volume budesonide rinses. All antibiotics were ceased 30 days prior to recruitment. Only saline irrigations was allowed, both as a rescue medication and to control for the potentially beneficial effects of saline therapy used as vehicle for the bacteria.

Approval was obtained from Health Canada for intranasal administration of live *L. lactis W136* bacteria (Health Canada registration number: 191920) and the CHUM Institutional Review Board and Ethics committee (Registration No. 12.288) prior to trial performance. *L. lactis W136* was furnished free of charge by Agropur Dairy Cooperative (St Hubert, QC, Canada). Patients were drawn from ongoing clinical activities, and consent obtained by a member of the study team not involved in clinical care of the patients. No financial incentive was prided for participation, apart from reimbursement of hospital parking expenses. All measures were obtained and processed ensuring patient data protection and confidentiality.

**Figure 2:**
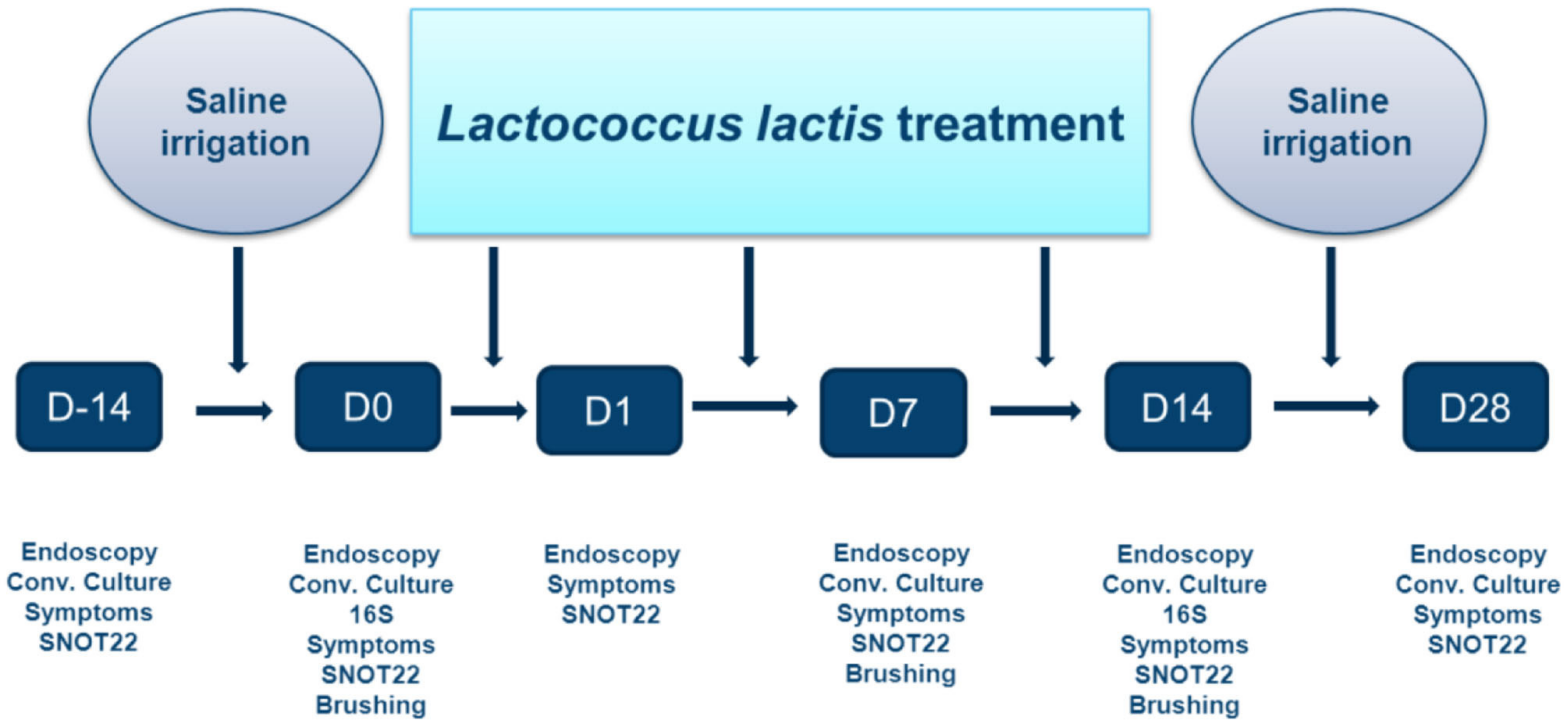
Timeline of probiotic trial. Study protocol showing initial 14 days saline-only washout period; 14 days of *L. lactis W136* followed by 14 day saline-only observational period (D, Day).

The treatment protocol is outlined in Figure 2. Following recruitment, a 2-week washout period occurred during which all sinus medications were ceased, save for nasal and sinus irrigation with 120 mL of 0.9% saline solution administered twice-daily using the NeilMed Pediatric Sinus Rinse system (NeilMed Pharmaceuticals Inc., Santa Rosa, CA). Subjects were then treated with 14 days of BID irrigations containing 1.2 × 10^9^ colony forming units (CFU) of live *L. lactis W136* suspended in 120 mL 0.9% buffered saline (10^7^ CFU/mL concentration). This was followed by a second 14-day period during where only the irrigation with saline was continued. For each application, 120 ml (pediatric size) of clean water was mixed with the appropriate NeilMed SinusRinse pediatric packet, and the frozen *L. lactis* added. If the pellet was difficult to dislodge from the Eppendorf tube, a drop or two of saline form the NeilMed bottle was put in the Eppendorf to dissolve it. Patients shook the bottle, and then rinsed their nose over the sink. The head was held at a 45° angle and the irrigation fluid irrigated through one nostril and out the other. Treatment was done until no product was left. Technique was demonstrated to the patient and the first irrigation was performed under direct observation. A new bottle was supplied for every rinsing to avoid cleaning the bottle in between uses.

*Lactococcus lactis W136* was provided to subjects in frozen format for reconstitution in single-dose containers. *L. lactis W136* was reconstituted immediately prior to administration in 120 mL of buffered saline 0.9% solution using a 120 mL nasal irrigation device. A new irrigation bottle was supplied for each application. The first application was administered in clinic under supervision.

Assessments were performed at the beginning and end of the washout period, at the initiation of *L. lactis W136* treatment (day 0), and at day 1, 7, 14, and 28 after initiation of the tested therapy. Symptomatology was assessed using total sinonasal symptom score (SNSS), validated disease-specific quality of life questionnaire; Sino-nasal Outcome Test 22 item (SNOT-22) (Hopkins et al., 2009) and aspect of the sinus mucosa as assessed by a validated endoscopy score (POSE score) (Wright and Agrawal, 2007). Additional monitoring for safety included monitoring: (i) sense of smell using the UPSIT-40 (Doty et al., 1984) (40-item University of Pennsylvania Smell Identification Test (Sensonics, Inc., Haddon Heights, NJ), (ii) Possible adverse effects on middle ear and Eustachian tube function by monitoring middle ear status using otomicroscopy to improve diagnostic accuracy, (iii) endoscopically-obtained swab cultures (COPAN, Becton & Dickinson Company, Sparks, MD, USA) to identify overgrowth by pathogenic or probiotic bacteria. Safety was further assessed by monitoring adverse events.

Samples were collected by standard culture swab for routine culture and a nasal brushing for 16S microbiome analysis obtained at Do, prior to first probiotic application, treatment, and at D14, the day following the last treatment. All probiotic irrigations were performed at least 12 h prior to the D14 visit.

Samples for 16S were processed at the Surette Laboratory (Hamilton, ON, Canada) where DNA extraction, 16S rRNA gene amplification and sequencing was performed. DNA was extracted from the whole swabs and its concentration measured with a Nanodrop 2000c Spectrophotometers (Fisher Scientific, Hampton, NH, USA). The quality of the extracted DNA was evaluated on a 1% agarose gel. Libraries were prepared by amplifying the V3 hypervariable region of the 16S rRNA gene based on a modified version of the libraries described by Bartram et al. (2011). Primers used were GC-341F (5′-CCTACGGGAGGCAGCAG-3′) and 518R (5′-ATTACCGCGGCTGCTG-3′). Amplicons were amplified by PCR and normalized according to the obtained concentrations prior to sequencing. The MiSeq platform was used for 250 bp paired-end sequencing of PCR products.

### Statistical Analysis

Response to treatment was determined by calculating time-weighted average scores over the study period from day 0 to day 28, using “time” as day 0 as the baseline value. Results were expressed in term of confidence intervals (CI).

### Microbiome Analysis

Sequence reads were processed and annotated using the ANCHOR pipeline (Gonzalez et al., 2019). Briefly, sequences were aligned and dereplicated before selection of OTUs using a count threshold of 14 across all samples. Annotation queried four sequence repositories with strict BLASTn criteria (>99% identity and coverage): NCBI curated bacterial and Archaea RefSeq, NCBI nt, SILVA, and Ribosomal Database Project (RDP). When the highest identity/coverage was shared amongst multiple different (Gonzalez et al., 2018) putative annotation, all were retained and reported; borrowed from the idea of secondary annotation in metatranscriptomics. Amplicons with low-counts (<14) were binned to high-count sequences in a second BLASTn, using a lower threshold of >98% identity/coverage (secondary count capture). Alpha diversity was measured using Shannon and inverse Simpson indices within Phyloseq R package (McMurdie and Holmes, 2013). Beta diversity was estimated using Bray-Curtis dissimilarity and the Constrained Analysis of Principal Coordinates (CAP) ordination method. Dispersion ellipses were drawn using veganCovEllipse function from Vegan package in R (Oksanen et al., 2016). Significant distance was evaluated between the groups using non-parametric analysis of similarities (ANOSIM) on normalized counts based on Bray distances (R Vegan package). Differential abundance analysis on 16S rRNA gene amplicons was performed using DESeq2 (Love et al., 2014), which can perform well with uneven library sizes and sparsity common to 16S rRNA gene data (Weiss et al., 2017; Gonzalez et al., 2018; Minerbi et al., 2019). A differential abundance selection parameter of false discovery rate (FDR; Benjamini-Hochberg procedure) <0.05 was applied. Raw counts were transformed using regularized log transformation across samples (rlog function, R phyloseq package).

## Results

Twenty-seven patients who met inclusion/exclusion criteria were recruited. One patient was withdrawn before initiation of the trial because of scheduling issues, and two other patients withdrawn during the saline treatment period as their symptoms became intolerable following withdrawal of usual sinus medication during run-in. None of these patients received *L. lactis W136* (Figure 1).

**Figure 3:**
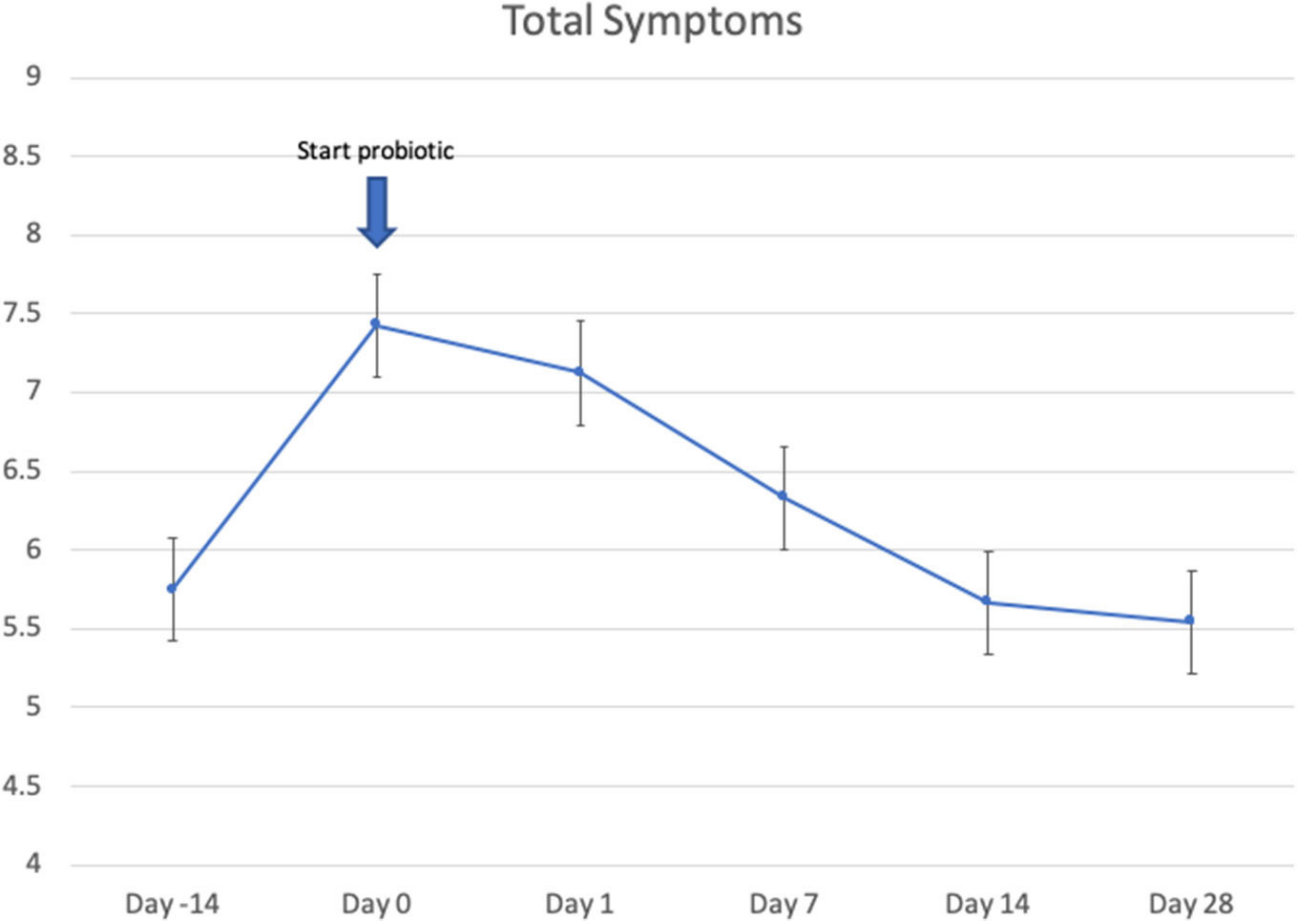
Effect of *L. lactis W136* on total nasal symptom score (*n* = 24). Time-weighted average scores over the study period from day 0 (D0) to day 28 (D28).

**Figure 4:**
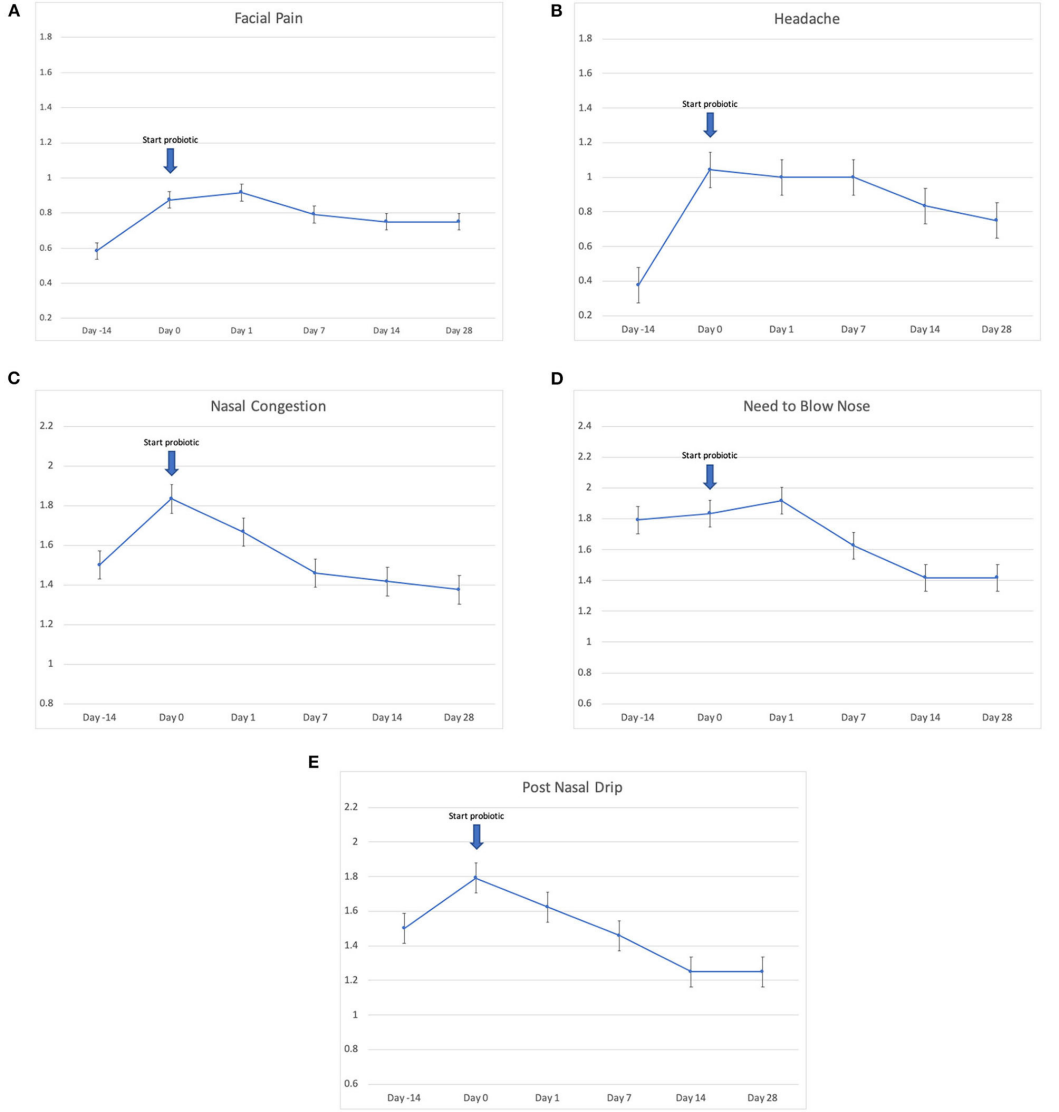
Effect of *L. lactis W136* on individual nasal symptoms (0–3 scale). **(A)** Facial pain; **(B)** headache; **(C)** nasal congestion; **(D)** need to blow nose; and **(E)** post-nasal drip (*n* = 24). Time-weighted average scores over the study period from day 0 (D0) to day 28 (D28).

**Figure 5:**
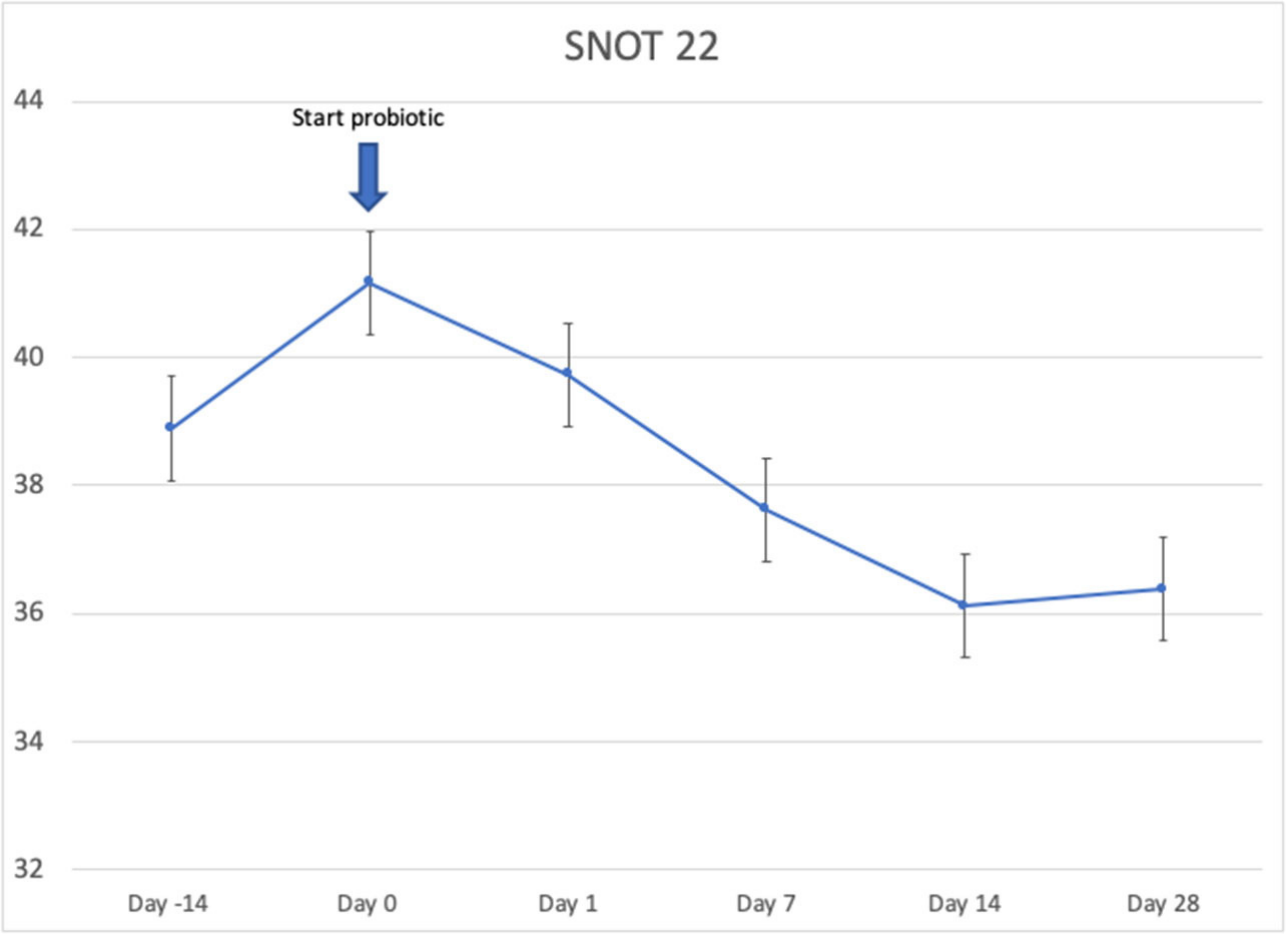
Effect of *L. lactis W136* on SNOT-22. Mean of total score of the 22 items from quality of life questionnaire (*n* = 24). Time-weighted average scores over the study period from day 0 (D0) to day 28 (D28).

**Figure 6:**
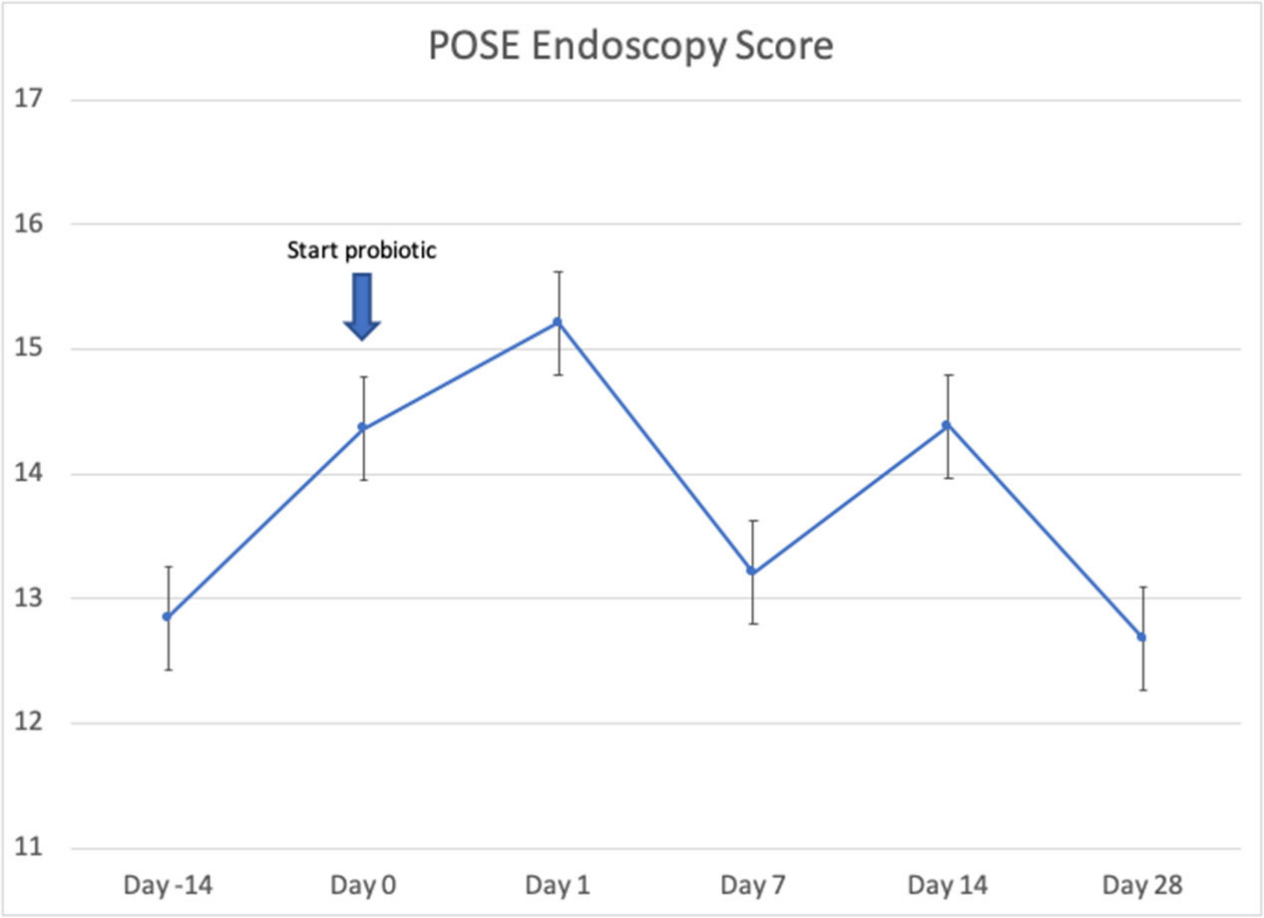
Effect of *L Lactis W136* on sinus endoscopy: Total POSE score. (*n* = 24).

**Figure 7:**
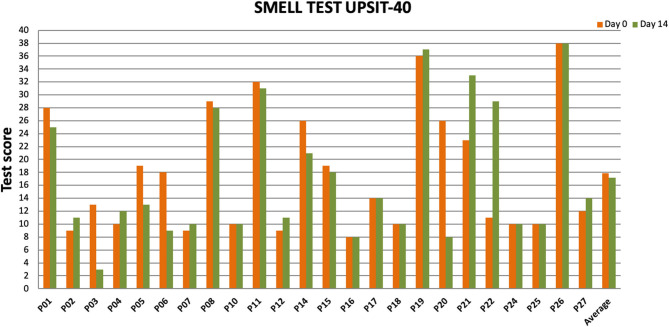
Impact of *L. lactis W136* treatment on olfaction. UPSIT-40 scores prior to (Day 0) and immediately following treatment (Day 14) presented individually for all patients (*n* = 24) (D, Day).

Twenty-four patients received *L. lactis W136*, and all 24 successfully completed the study. Their baseline characteristics are summarized in Table 1. Overall, 14 days of treatment with *L. lactis W136* was well-tolerated and all 24 patients were able to complete a full course. No acute infections occurred. The saline only washout period was associated with a deterioration in most parameters, with increased symptoms, decreased disease specific QOL, and deteriorated mucosal aspect. Following initiation of treatment, symptoms progressively improved over the subsequent 28-day treatment and observation period (Mean change = 6.0; 95% CI: 4.65–7.36) (Figure 3). Pattern of response showed progressive improvement during the 14 days of *L. lactis W136* administration, with improvements maintained over the 14-day post-treatment observation period. Individual symptoms of facial pain, headache, nasal congestion, need to blow nose and post-nasal drip all followed a similar pattern of improvement (Figure 4). Symptoms showing greatest magnitudes of improvement were for the symptoms of “Nasal congestion” (95% CI: 1.10–1.81), “Post-nasal drip” (95% CI: 1.04–1.67) and “Need to blow nose” (95% CI: 1.26–1.81). SNOT-22 scores followed a similar pattern as for symptomatology. SNOT-22 score significantly improved over the course of the trial (95% CI: 27.28–46.87). Again, improvement persisted following administration of probiotic, and was maintained at the 28-day point (Figure 5). Mucosal aspect as assessed by endoscopic score demonstrated a similar pattern of improvement as for sinus symptoms and QOL (95% CI: 10.32 to 16.28) (Figure 6). Sense of smell as assessed by UPSIT-40 scores remained stable during the trial, with no evidence of impairment from therapy (95% CI: −3.46 to 2.13) (Figure 7).

**Table 1:** Population baseline characteristics.

**CRSwNP/CRSsNP (*n*)**	**17/7**
Age (mean ± SD)	54.9 ± 11.9 years
Gender (*n*)	Female = 13 (54%)
Ethnicity (*n*)	22 (Caucasian); 2 (Arabic)
Asthma [*n* (%)]	18 (75%)
Number of previous ESS (mean ± SD)	2.08 ± 1.14
History of allergy (seasonal) [*n* (%)]	5 (21%)
Current smoker	0 (0%)
WBC (10^9^/L) (mean ± SD)	6.83 ± 1.29
Eos (10^9^/L) (mean ± SD)	0.39 ± 0.32
IgE kIU/L(mean ± SD)	275.8 ± 397.7

*n, number; SD, standard deviation*.

### Bacteriology and Microbiome Assessment

**Figure 8:**
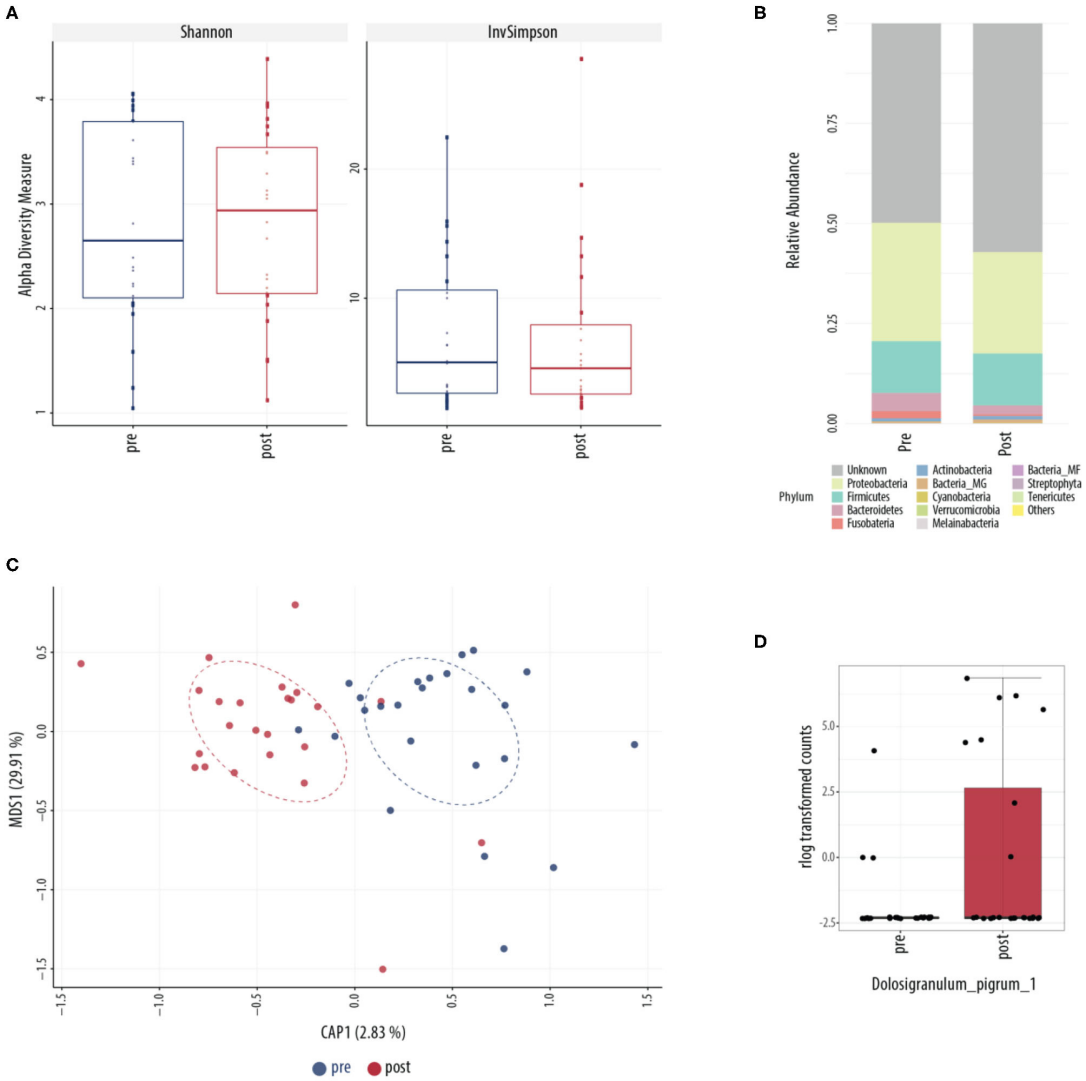
Sinus microbial analysis of patients before and after ESS. **(A)** A comparison of alpha diversity indices (Shannon and inverse Simpson). **(B)** Compared relative abundance (from raw counts). **(C)** Canonical analysis of principal coordinates of taxa abundance from raw counts. No significant differences between the groups were observed in alpha and beta diversities. **(D)** Differentially abundant OTUs (FDR < 0.05) between the pre-surgery (pre, *n* = 24) and post-surgery (post, *n* = 24) groups. The main axis represents the fold change (log_2_) in relative abundance of significantly different OTUs between the two groups and their normalized counts. Values represent mean score for all enrolled patients (*n* = 24). Change in microbiome is calculated from day 0 to day 14 period (D, Day, D0, introduction of probiotic; D14, end of treatment).

Conventional culture identified no noticeable change in patterns of collection or in type of bacteria collected over the course of treatment (Table 2). This was further explored using culture-independent 16S microbiome assessment. Samples were able to be collected from all 24 patients. An average of 82,347 (range between 15,352 and 347,360) counts per sample (*N* = 48) were obtained grouped into 6,820 OTUs. Following *L. lactis W136* administration, there was no change in alpha diversity between both sample groups (Figure 8A). Phylum analysis (Figure 8B) suggested differences in microbiome composition, however CAP plots showed no major differences (Figure 8C). Differential abundance analysis using DESeq2 showed a significant decrease in the abundance of *Dolosigranulum pigrum* in post-therapy patients (Figure 8D).

**Table 2:** Bacteriology by conventional culture over the trial period.

	**D-14 (%)**	**D0 (16S) (%)**	**D7 (%)**	**D14 (16S) (%)**	**D28**	***p*-value**
**Culture** **+** **rate**	100.0	83.3	95.7	95.8	100.0	NS
Oropharyngeal flora	45.8	45.8	34.8	45.8	54.2	NS
*Corynebacterium* spp.	4.2	0.0	4.3	4.2	4.2	NS
*Coagulase-negative staphylococci*	25.0	8.3	21.7	20.8	8.3	NS
*Staphylococcus aureus*	37.5	20.8	39.1	37.5	37.5	NS
*Streptococcus pneumoniae*	4.2	4.2	0.0	0.0	12.5	NS
*Haemophilus influenzae*	4.2	0.0	4.3	4.2	0.0	NS
*Pseudomonas aeruginosa*	29.2	33.3	30.4	33.3	33.3	NS
*Enterobacter cloacae*	4.2	0.0	0.0	0.0	0.0	NS
*Escherichia coli*	4.2	4.2	8.7	8.3	8.3	NS
*Pseudomonas putida*	4.2	0.0	4.3	4.2	4.2	NS
*Proteus mirabilis*	4.2	4.2	4.3	4.2	0.0	NS
*Serratia marcescens*	4.2	4.2	0.0	0.0	0.0	NS
*Alternaria* spp.	4.2	0.0	0.0	0.0	0.0	NS

*There was no overgrowth of new pathogenic species and no growth of L. lactis on conventional culture before or after administration of L. lactis W136*.

### CRSwNP vs. CRSsNP Sub-Group Analysis

Exploratory analysis of CRSwNP and CRSsNP phenotypes was performed to verify comparability of groups at baseline and response to *L. lactis W136* administration. There was no significant difference at baseline scores for symptoms, SNOT-22, and endoscopy, nor was there any difference in response. The sole exception to this was the greater presence of polyps, as expected, in the polyp group (CRSwNP). There was however a difference between bacteria suggested by conventional culture results between the CRSwNP and CRSsNP groups (Table 3).

**Table 3:** Conventional culture subgroup analysis according to CRSsNP and CRSwNP phenotypes.

**Bacterial isolate**	**CRSsNP D-14 (%)**	**CRSwNP D-14 (%)**	**All D-14 (%)**	***t*-test CRSwNP vs. CRSsNP**
*Oropharyngeal flora*	29	53	46	0.30
*Corynebacterium* ssp.	14	0	4	0.36
*Coagulase-negative staphylococci*	14	29	25	0.42
*Staphylococcus aureus*	57	29	38	0.26
*Streptococcus pneumoniae*	14	0	4	0.36
*Haemophilus influenzae*	14	0	4	0.36
*Pseudomonas aeruginosa*	43	24	29	0.42
*Enterobacter cloacae complex*	0	6	4	0.33
*Escherichia coli*	0	6	4	0.33
*Proteus mirabilis*	14	0	4	0.36
*Pseudomonas putida*	0	6	4	0.33
*Serratia marcescens*	0	6	4	0.33
*Alternaria* ssp.	0	6	4	0.33

*T-test compares CRSsNP and CRSwNP groups*.

**Figure 9:**
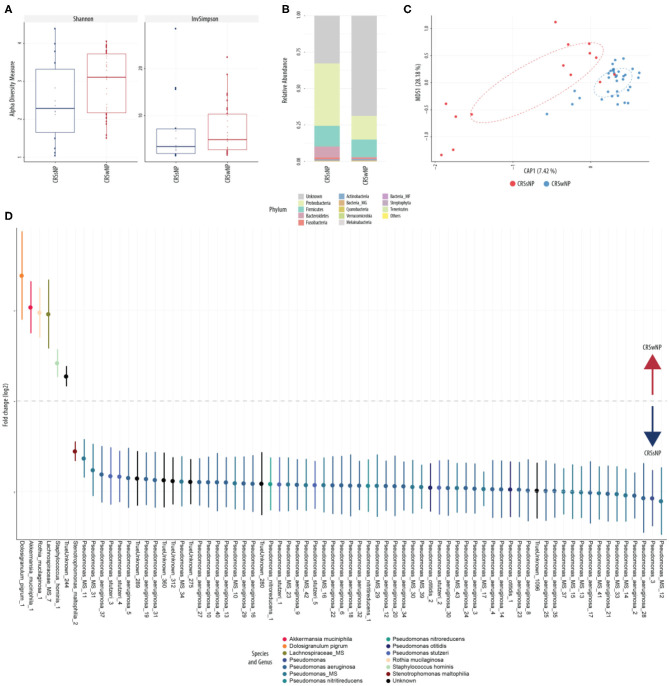
Sinus microbial analysis of patients without and with nasal polyps (CRSsNP, *n* = 7; CRSwNP, *n* = 17). **(A)** Comparison of alpha diversity indices (Shannon and inverse Simpson). **(B)** Comparison of relative abundance (from raw counts). **(C)** Canonical analysis of principal coordinates of taxa abundance from raw counts. Significant differences between the groups was observed in beta diversities (PERMANOVA, *p* < 0.01). **(D)** Differentially abundant OTUs (FDR < 0.05) between patients with polyps and patients without. The main axis represents the fold change (log_2_) in relative abundance of significantly different OTUs between the two groups and their normalized counts.

**Figure 10:**
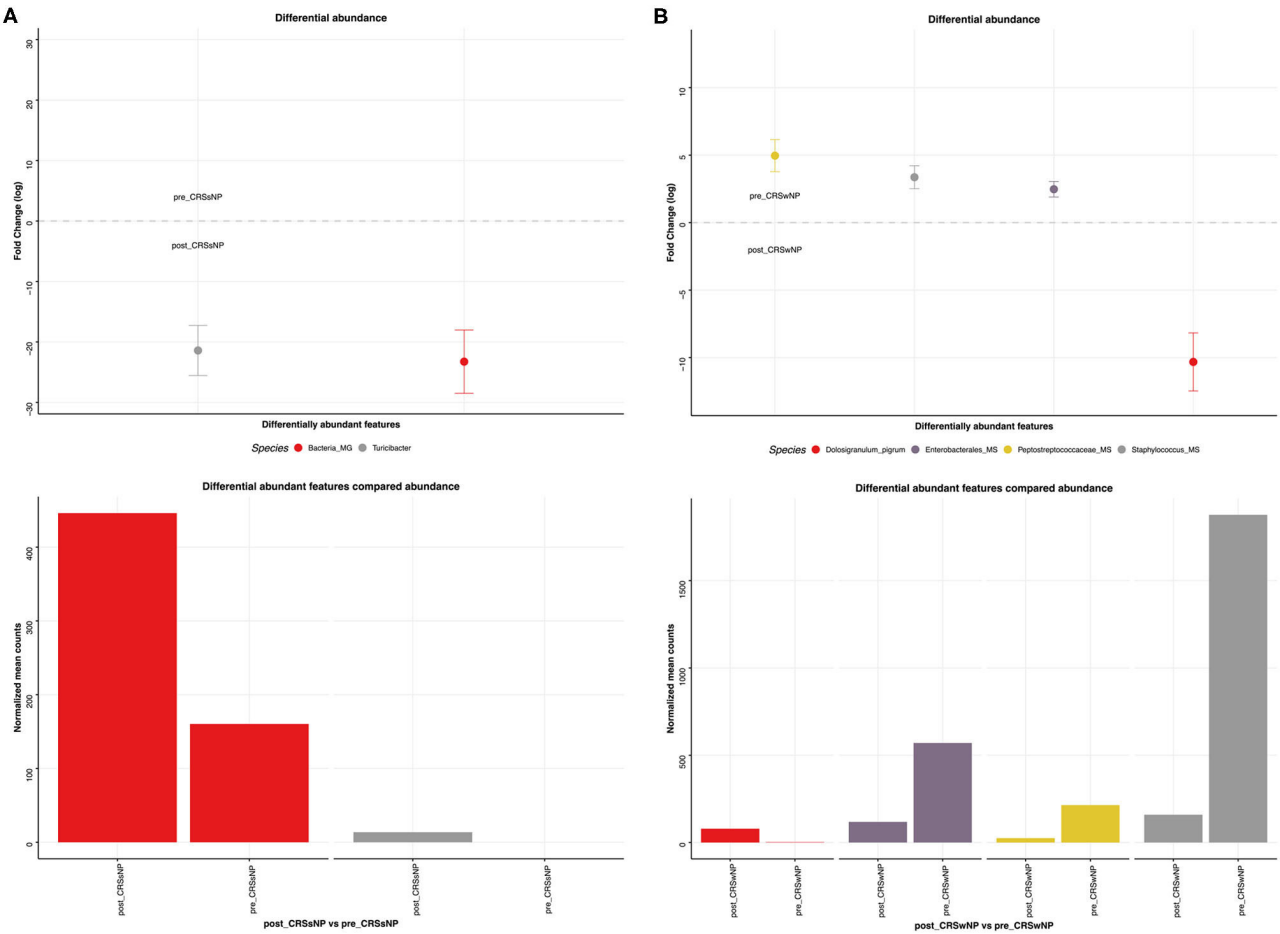
Sinus microbial analysis of microbiome modifications before and after *L. lactis W136* administration. **(A)** For CRSsNP (*n* = 7) and **(B)** For CRSwNP (*n* = 17) subgroups. Differentially abundant OTUs (FDR < 0.05) between before and after *L. lactis W136* administration. The main axis represents the fold change (log_2_) in relative abundance of significantly different OTUs between the two groups and their normalized counts.

This was explored further by assessing the microbiome composition at baseline and response to treatment separately for polyp and non-polyp subgroups. At baseline, differences in composition were seen between CRSsNP and CRSwNP groups (Figure 9). Notably, the CRSsNP group was characterized by a decrease in alpha diversity. Furthermore, at phylum level, these samples showed an increased relative abundance of *Proteobacteria*. When assessing differences at species level, the CRSsNP group demonstrated an increased abundance of *Dolosigranulum pigrum, Rothia mucilaginosa, Lachnospiraceae* spp., and *Staphylococcus hominis*. Differential abundance differences were also found. In fact, *Stenotrophomonas maltophilia*, multiple species of *Pseudomonas aeruginosa* and *Pseudomonas stutzeri* were found in higher proportion in CRSwNP patients. Changes associated with therapy were different for both groups (Figure 10). For CRSsNP, changes in microbiome was limited to the increase in *Turicibacter* spp., which was present only after treatment but at low levels. For CRSwNP, treatment was associated with reduced abundance of *Staphylococcus* MS (multiple species), *Peptostreptococcae* MS, *Enterobacialles* MS, and increased abundance of *Dolosigranulum pigrum*.

### Adverse Events

No serious adverse events occurred during this clinical trial (Table 4). Two patients showed evidence of middle ear effusion during the trial period, without symptoms or signs if acute infection. One patient had effusion and otorrhea already at time of inclusion. All patients reported ear fullness and ear pain in pre-treatment SNOT-22, suggesting pre-existing ET/middle ear disease. Effusion resolved completely in all patients without additional treatment at 1-month follow-up after end of study. Minor adverse events included: headache, migraines, nasal congestion, dental infection, throat pain, cold sore, gastroenteritis, nasal allergy, and shoulder pain; together all accounting for <4%. Some events were related to their condition of chronic rhinosinusitis and the other events pre-existed before the trial and medications were taken since before the trial.

**Table 4:** Adverse events occurring during study period.

**Adverse event**	**Frequency**	**Resolved at end of trial?**
Middle ear effusion (non-infectious)	2/24 (8.2%)	Y
Headache	1/24 (4.1%)	Y
Migraines	1/24 (4.1%)	Y
Nasal congestion	1/24 (4.1%)	Y
Dental infection	1/24 (4.1%)	Y
Throat pain	1/24 (4.1%)	Y
Cold sore	1/24 (4.1%)	Y
Gastroenteritis	1/24 (4.1%)	Y
Nasal allergy	1/24 (4.1%)	Y
Shoulder pain	1/24 (4.1%)	Y

*All events are recorded*.

## Discussion

This study offers further support that topical application of live bacteria directly to the nasal and sinus cavities via nasal irrigation administration is safe and well-tolerated in CRS patients refractory to previous medical and surgical therapy.

While administration of live bacteria to the sinuses is a relatively novel concept, this adds to existing evidence in animal and human models supporting that topical intranasal bacterial therapy is safe (Desrosiers et al., 2011; Cope and Lynch, 2015; Marchisio et al., 2015). Additionally, despite that this was not a placebo-controlled trial, during the period of administration of *L. lactis W136*, participants demonstrated improvements in their global sinus condition, as assessed by sinus symptoms, disease-specific quality of life indices, and endoscopically-assessed mucosal aspect of the sinuses. This was in marked contrast to the saline-only run-in period where symptoms and sinus endoscopy deteriorated rapidly.

The mechanisms by which the *L. lactis W136* could be exerting its effects in the study remains to be described, but mechanisms including modulation of immune responses and bacterial displacement of pathogenic species can be suspected from mechanisms previously identified in other disease models. *L. lactis* is a well-known probiotic bacterium, with immunomodulatory and antibacterial properties (Oelschlaeger, 2010). Immune modulation by lactic acid bacteria is well-described, the immunomodulatory effect exerted both via induction of IL-10 secretion (de Moreno de Leblanc et al., 2011) and silencing of TLR2 signaling (Fischer et al., 2011; Kaesler et al., 2016). For *L. lactis W136*, previous *in vitro* studies have confirmed that exposure to *L. lactis* increases IL-10 induction in a suspension of peripheral blood mononuclear cells (Moles et al., 2020).

Interference with other pathogens in the sinus cavities may also play a role. In this study, *Dolosigranulum pigrum* was seen in low abundance pre-treatment and was increased in abundance following *L. lactis W136* treatment. *D. pigrum* has only recently been identified in the microbiome of cystic fibrosis lungs (Lopes et al., 2017), where it is described as a pathogen with the capacity to form biofilms of large biomass which are resistant to conventional antibiotic therapy. Intriguingly, it may also have a role in regulation of other pathogens and is considered to play a key role in inflammatory disorders of the nose, nasopharynx, and paranasal sinuses (Brugger et al., 2016). Working alone, *in vitro* it is capable of inhibiting the growth of *S. aureus*, and, in combination with *Corynebacterium pseudodipthericum*, it is capable of inhibiting the growth of *Streptococcus pneumoniae* and *Haemophilus influenzae* (Brugger et al., 2020). *D. pigrum* has been suggested as a novel “probiotic” bacteria (Lappan and Peacock, 2019). However, direct administration of live *D. pigrum* will require careful assessment because of its potential for producing human disease, thus it may find a role as a marker of microbiome status than a therapeutic agent.

CRSwNP and CRSsNP phenotypes appeared to have different microbiome patterns, both at baseline and in terms of response to treatment. At baseline, CRSsNP appears to have an over-abundance *Pseudomonas* spp. mainly including *Pseudomonas aeruginosa*, while in CRSwNP, other species predominate. Apart from the lower relative abundance of *D. pigrum* in the CRSsNP group, it is interesting to note the low relative abundance of *Akkermansia muciniphila* in the CRSsNP group. Reduced levels of *A. muciniphila* are associated with obesity, diabetes, cardiometabolic diseases, low-grade inflammation (Cani and de Vos, 2017), and lesser responses to PD-1 inhibitors for cancer (Routy et al., 2018). Supplementation with *A. mucinophilia* as a novel probiotic has recently been proposed (Zhou and Zhang, 2019).

Overall, these microbiome differences may reflect differing mechanisms of disease development which create unique microenvironments selecting for dysbiotic flora. Changes in the microbiome associated with *L. lactis 136* treatment differ between these two entities. CRSsNP microbiomes were not greatly impacted by administration of *L. lactis W136*. This is consistent with *in vitro* observations where co-culture of *L. lactis W136* with clinical isolates of *P. aeruginosa* did not have an effect (Cho et al., 2020). While *L. lactis* administration is associated with an increase in *Turicibacter*, a bacterium implicated in serotonin metabolism (Fung et al., 2019), its abundance here is so low it is difficult to believe it is playing a great role in disease causation or prevention. In CRSwNP, increased abundance of *D. pigrum* following *L. lactis W136* treatment suggests an unexpected protective role for this bacterium.

These observations emphasize that the effects of microbiome supplementation may thus be more complex than direct associations suggested by *in vitro* susceptibility testing methods. Instead of exerting their effects directly, bacteria may interact with the mucosal barrier or mucosal immunity to compete for the ecological niche that then effect the observed changes. In addition, apparently different patterns of changes in the microbiome for CRSwNP and CRSsNP suggest these might be best considered as distinct entities for future assessments. This may contribute to different responses during interaction with *L. lactis W136* and suggest that future trials consider these entities separately.

The occurrence of middle ear effusion in two subjects is worthy of further discussion. The mechanism of development of ear effusion is unknown but may be related to other factors than probiotic administration, such as (i) deterioration of pre-existing Eustachian tube dysfunction and/or upper respiratory tract disease following withdrawal of therapy or (ii) performance of nasal irrigation. ET dysfunction is frequent in CRS patients particularly in those with severe disease (Stoikes and Dutton, 2005; Maniakas et al., 2018) and may deteriorate following withdrawal of CRS medication. This concept is supported by a recent clinical trial of 60 patients treated with Dupilumab or placebo for CRSwNP where otitis media developed in a patient in the untreated placebo group treated only saline irrigation (Bachert et al., 2016).

### Study Limitations

This pilot study is not a parallel group-controlled trial, but rather a feasibility and safety study to better understand the feasibility of this therapeutic approach. We understand this is a limitation but, the placebo or therapeutic effects of vehicle administration (saline) have been controlled for by using saline irrigations during the run-in period while all other sinus medications were ceased, thus we feel that effects observed following initiation of therapy are secondary to the latter and not the saline irrigation. The effect of saline irrigation alone in severe CRS is expected to be limited. This is confirmed by the aggravation of symptoms and deterioration of QOL and mucosal aspect following withdrawal of medications during the run-in period. In addition, saline irrigations have been shown not to influence microbiome composition in CRS patients (Liu et al., 2015). That *L. lactis W136* was associated with a time-dependent improvement suggests it may be having an effect, possibly via one of the mechanisms postulated above.

## Conclusion

Topical intranasal administration of live *L. lactis W136* for 14 days in patients with chronic rhinosinusitis refractory to medical and surgical therapy was well-tolerated, without serious adverse events or new infections. Improvements noted in symptoms, quality of life, and mucosal aspect suggest that topical administration of *Lactococcus lactis W136* may potentially represent novel alternative therapy for patients with sinus disease, making it worthy of further investigation.

## Data Availability Statement

The original contributions presented in the study are publicly available. This data can be found here: https://www.ncbi.nlm.nih.gov/sra/PRJNA638899.

## Ethics Statement

The studies involving human participants were reviewed and approved by CHUM Institutional Review Board and Ethics committee (Registration No. 12.288). The patients/participants provided their written informed consent to participate in this study.

## Author Contributions

LE, SA, JM, BC, and MD contributed conception and design of the study. LE, SA, EG, and AR organized the database. EG performed the statistical analysis. MD wrote the first draft of the manuscript. LE, SA, EG, AR, and MD wrote sections of the manuscript. All authors contributed to the manuscript revision, read, and approved the submitted version.

## Conflict of Interest

MD is the founder of Probionase Therapies Inc., a start-up company founded following the conclusion of this trial to develop probiotic bacteria for therapeutic purposes. The remaining authors declare that the research was conducted in the absence of any commercial or financial relationships that could be construed as a potential conflict of interest.

## Abbreviations

Eos: eosinophils
CFU: colony-forming units
CI: confidence interval
CRS: chronic rhinosinusitis
CRSsNP: chronic rhinosinusitis without nasal polyps
CRSwNP: chronic rhinosinusitis with nasal polyps
ESS: endoscopic sinus surgery
ET: Eustachian tube
IgE: immunoglobulin E
L. lactis W136: Lactococcus lactis W136
MCID: minimal clinically important difference
POSE: peri-operative sinus endoscopy
QOL: quality of life
S aureus: Staphylococcus aureus
SNSS: sino-nasal symptom score
SNOT-22: Sino-nasal Outcome Test 22 items
UPSIT-40: University of Pennsylvania Smell Identification Test 40-items
WBC: white blood cells.

## References

[B1] Abreu N. A. Nagalingam N. A. Song Y. Roediger F. C. Pletcher S. D. Goldberg A. N.. (2012). Sinus microbiome diversity depletion and Corynebacterium tuberculostearicum enrichment mediates rhinosinusitis. Sci. Transl. Med. 4:151ra124. 10.1126/scitranslmed.3003783 22972842 PMC4786373

[B2] Al-Shemari H. Abou-Hamad W. Libman M. Desrosiers M. (2007). Bacteriology of the sinus cavities of asymptomatic individuals after endoscopic sinus surgery. J. Otolaryngol. 36, 43–48. 10.2310/7070.2006.0019 17376350

[B3] Aroniadis O. C. Brandt L. J. (2013). Fecal microbiota transplantation: past, present and future. Curr. Opin. Gastroenterol. 29, 79–84. 10.1097/MOG.0b013e32835a4b3e 23041678

[B4] Bachert C. Mannent L. Naclerio R. M. Mullol J. Ferguson B. J. Gevaert P.. (2016). Effect of subcutaneous dupilumab on nasal polyp burden in patients with chronic sinusitis and nasal polyposis: a randomized clinical trial. JAMA 315, 469–479. 10.1001/jama.2015.19330 26836729

[B5] Bartram A. K. Lynch M. D. Stearns J. C. Moreno-Hagelsieb G. Neufeld J. D. (2011). Generation of multimillion-sequence 16S rRNA gene libraries from complex microbial communities by assembling paired-end illumina reads. Appl. Environ. Microbiol. 77, 3846–3852. 10.1128/AEM.02772-10 21460107 PMC3127616

[B6] Bjerg A. T. Sorensen M. B. Krych L. Hansen L. H. Astrup A. Kristensen M.. (2015). The effect of *Lactobacillus paracasei* subsp. paracasei *L. casei* W8(R) on blood levels of triacylglycerol is independent of colonisation. Benef. Microbes 6, 263–269. 10.3920/BM2014.0033 25273547

[B7] Brahe L. K. Le Chatelier E. Prifti E. Pons N. Kennedy S. Blædel T.. (2015). Dietary modulation of the gut microbiota–a randomised controlled trial in obese postmenopausal women. Br. J. Nutr. 114, 406–417. 10.1017/S0007114515001786 26134388 PMC4531470

[B8] Brugger S. D. Bomar L. Lemon K. P. (2016). Commensal–Pathogen Interactions along the Human Nasal Passages. PLOS Pathogens 12:e1005633. 10.1371/journal.ppat.1005633 27389401 PMC4936728

[B9] Brugger S. D. Eslami S. M. Pettigrew M. M. Escapa I. F. Henke M. T. Kong Y.. (2020). Dolosigranulum pigrum cooperation and competition in human nasal microbiota. bioRxiv. 678698. 10.1101/678698 32907957 PMC7485692

[B10] Cani P. D. de Vos W. M. (2017). Next-generation beneficial microbes: the case of *Akkermansia muciniphila*. Front. Microbiol. 8:1765. 10.3389/fmicb.2017.01765 29018410 PMC5614963

[B11] Chalermwatanachai T. Vilchez-Vargas R. Holtappels G. Lacoere T. Jáuregui R. Kerckhof F. M.. (2018). Chronic rhinosinusitis with nasal polyps is characterized by dysbacteriosis of the nasal microbiota. Sci. Rep. 8:7926. 10.1038/s41598-018-26327-2 29784985 PMC5962583

[B12] Cho S.-T. Kung H.-J. Huang W. Hogenhout S. A. Kuo C.-H. (2020). Species boundaries and molecular markers for the classification of 16SrI phytoplasmas inferred by genome analysis. Front. Microbiol. 11:1531. 10.3389/fmicb.2020.01531 32754131 PMC7366425

[B13] Cleland E. J. Drilling A. Bassiouni A. James C. Vreugde S. Wormald P. J. (2014). Probiotic manipulation of the chronic rhinosinusitis microbiome. Int. Forum Allergy Rhinol. 4, 309–314. 10.1002/alr.21279 24415658

[B14] Cope E. K. Lynch S. V. (2015). Novel microbiome-based therapeutics for chronic rhinosinusitis. Curr. Allergy Asthma Rep. 15:504. 10.1007/s11882-014-0504-y 25777787

[B15] de Moreno de Leblanc A. Del Carmen S. Zurita-Turk M. Rocha C. S. van de Guchte M. Azevedo V.. (2011). Importance of IL-10 modulation by probiotic microorganisms in gastrointestinal inflammatory diseases. ISRN Gastroenterol. 2011:892971. 10.5402/2011/892971 21991534 PMC3168568

[B16] Desrosiers M. Evans G. A. Keith P. K. Wright E. D. Kaplan A. Bouchard J. (2011). Canadian clinical practice guidelines for acute and chronic rhinosinusitis. J. Otolaryngol. Head Neck Surg. 40(Suppl. 2):S99–193. 10.1186/1710-1492-7-2 21658337

[B17] Doty R. L. Shaman P. Dann M. (1984). Development of the University of Pennsylvania Smell Identification Test: a standardized microencapsulated test of olfactory function. Physiol. Behav. 32, 489–502. 10.1016/0031-9384(84)90269-5 6463130

[B18] Ferrario C. Taverniti V. Milani C. Fiore W. Laureati M. De Noni I.. (2014). Modulation of fecal Clostridiales bacteria and butyrate by probiotic intervention with *Lactobacillus paracasei* DG varies among healthy adults. J. Nutr. 144, 1787–1796. 10.3945/jn.114.197723 25332478

[B19] Fischer K. Stein K. Ulmer A. J. Lindner B. Heine H. Holst O. (2011). Cytokine-inducing lipoteichoic acids of the allergy-protective bacterium *Lactococcus lactis* G121 do not activate via Toll-like receptor 2. Glycobiology 21, 1588–1595. 10.1093/glycob/cwr071 21666273

[B20] Fung T. C. Vuong H. E. Luna C. D. G. Pronovost G. N. Aleksandrova A. A. Riley N. G.. (2019). Intestinal serotonin and fluoxetine exposure modulate bacterial colonization in the gut. Nat. Microbiol. 4, 2064–2073. 10.1038/s41564-019-0540-4 31477894 PMC6879823

[B21] Gonzalez E. Pitre F. E. Brereton N. J. B. (2019). ANCHOR: a 16S rRNA gene amplicon pipeline for microbial analysis of multiple environmental samples. Environ. Microbiol. 21, 2440–2468. 10.1111/1462-2920.14632 30990927 PMC6851558

[B22] Gonzalez E. Pitre F. E. Pagé A. P. Marleau J. Guidi Nissim W. St-Arnaud M.. (2018). Trees, fungi and bacteria: tripartite metatranscriptomics of a root microbiome responding to soil contamination. Microbiome 6:53. 10.1186/s40168-018-0432-5 29562928 PMC5863371

[B23] Hopkins C. Gillett S. Slack R. Lund V. J. Browne J. P. (2009). Psychometric validity of the 22-item Sinonasal Outcome Test. Clin. Otolaryngol. 34, 447–454. 10.1111/j.1749-4486.2009.01995.x 19793277

[B24] Kaesler S. Skabytska Y. Chen K. M. Kempf W. E. Volz T. Köberle M.. (2016). Staphylococcus aureus-derived lipoteichoic acid induces temporary T-cell paralysis independent of Toll-like receptor 2. J. Allergy Clin. Immunol. 138, 780.e6–790.e6. 10.1016/j.jaci.2015.11.043 26949056

[B25] Kristensen N. B. Bryrup T. Allin K. H. Nielsen T. Hansen T. H. Pedersen O. (2016). Alterations in fecal microbiota composition by probiotic supplementation in healthy adults: a systematic review of randomized controlled trials. Genome Med. 8:52. 10.1186/s13073-016-0300-5 27159972 PMC4862129

[B26] Lai Y. Di Nardo A. Nakatsuji T. Leichtle A. Yang Y. Cogen A. L.. (2009). Commensal bacteria regulate Toll-like receptor 3-dependent inflammation after skin injury. Nat. Med. 5, 1377–1382. 10.1038/nm.2062 19966777 PMC2880863

[B27] Lappan R. Peacock C. S. (2019). Corynebacterium and Dolosigranulum: future probiotic candidates for upper respiratory tract infections. Microbiol. Aust. 40, 172–177. 10.1071/MA19051

[B28] Liu C. M. Kohanski M. A. Mendiola M. Soldanova K. Dwan M. G. Lester R.. (2015). Impact of saline irrigation and topical corticosteroids on the postsurgical sinonasal microbiota. Int. Forum Allergy Rhinol. 5, 185–190. 10.1002/alr.21467 25556553 PMC4628778

[B29] Lopes S. P. Azevedo N. F. Pereira M. O. (2017). Developing a model for cystic fibrosis sociomicrobiology based on antibiotic and environmental stress. Int. J. Med. Microbiol. 307, 460–470. 10.1016/j.ijmm.2017.09.018 29033313

[B30] Love M. I. Huber W. Anders S. (2014). Moderated estimation of fold change and dispersion for RNA-seq data with DESeq2. Genome Biol. 15:550. 10.1186/s13059-014-0550-8 25516281 PMC4302049

[B31] Maniakas A. Desrosiers M. Asmar M. H. Al Falasi M. Endam L. M. Hopkins C.. (2018). Eustachian tube symptoms are frequent in chronic rhinosinusitis and respond well to endoscopic sinus surgery. Rhinology 56, 118–121. 10.4193/Rhin17.165 29509831

[B32] Marchisio P. Santagati M. Scillato M. Baggi E. Fattizzo M. Rosazza C.. (2015). *Streptococcus salivarius* 24SMB administered by nasal spray for the prevention of acute otitis media in otitis-prone children. Eur. J. Clin. Microbiol. Infect. Dis. 34, 2377–2383. 10.1007/s10096-015-2491-x 26385346

[B33] McMurdie P. J. Holmes S. (2013). phyloseq: an R package for reproducible interactive analysis and graphics of microbiome census data. PLoS ONE. 8:E61217. 10.1371/journal.pone.0061217 23630581 PMC3632530

[B34] Meltzer E. O. Hamilos D. L. Hadley J. A. Lanza D. C. Marple B. F. Nicklas R. A.. (2004). Rhinosinusitis: establishing definitions for clinical research and patient care. Otolaryngol. Head Neck Surg. 131(Suppl. 6), S1–S62. 10.1016/j.otohns.2004.09.067 15577816 PMC7118860

[B35] Minerbi A. Gonzalez E. Brereton N. J. B. Anjarkouchian A. Dewar K. Fitzcharles M. A.. (2019). Altered microbiome composition in individuals with fibromyalgia. Pain 160, 2589–2602. 10.1097/j.pain.0000000000001640 31219947

[B36] Moles L. Gómez M. Moroder E. Bustos G. Melgar A. Del Campo R.. (2020). *Staphylococcus epidermidis* in feedings and feces of preterm neonates. PLoS ONE 15:e0227823. 10.1371/journal.pone.0227823 32012172 PMC6996929

[B37] Nader M. E. Abou-Jaoude P. Cabaluna M. Desrosiers M. (2010). Using response to a standardized treatment to identify phenotypes for genetic studies of chronic rhinosinusitis. J. Otolaryngol. Head Neck Surg 39, 69–75. 20122348

[B38] Oelschlaeger T. A. (2010). Mechanisms of probiotic actions - a review. Int. J. Med. Microbiol. 300, 57–62. 10.1016/j.ijmm.2009.08.005 19783474

[B39] Oksanen J. Blanchet F. G. Friendly M. Kindt R. Legendre P. McGlinn D. (2016) vegan: Community Ecology Package. R package version 2.4-3. Vienna: R Foundation for Statistical Computing. [Google Scholar].

[B40] Routy B. Le Chatelier E. Derosa L. Duong C. P. M. Alou M. T. Daillère R.. (2018). Gut microbiome influencesefficacy of PD-1-based immunotherapy against epithelial tumors. Science 359, 91–97. 10.1126/science.aan3706 29097494

[B41] Schwartz J. S. Peres A. G. Mfuna Endam L. Cousineau B. Madrenas J. Desrosiers M. (2016). Topical probiotics as a therapeutic alternative for chronic rhinosinusitis: a preclinical proof of concept. Am. J. Rhinol. Allergy 30, 202–205. 10.2500/ajra.2016.30.4372 28124641

[B42] Snelling A. M. (2005). Effects of probiotics on the gastrointestinal tract. Curr. Opin. Infect. Dis. 18, 420–426. 10.1097/01.qco.0000182103.32504.e3 16148529

[B43] Song A. A. In L. L. A. Lim S. H. E. Rahim R. A. (2017). A review on *Lactococcus lactis*: from food to factory. Microb. Cell Fact. 16:55 10.1186/s12934-017-0754-1 28376880 PMC5379754

[B44] Stephenson M. F. Mfuna L. Dowd S. E. Wolcott R. D. Barbeau J. Poisson M.. (2010). Molecular characterization of the polymicrobial flora in chronic rhinosinusitis. J. Otolaryngol. Head Neck Surg. 39, 182–187. 20211106

[B45] Stjarne P. Olsson P. Alenius M. (2009). Use of mometasone furoate to prevent polyp relapse after endoscopic sinus surgery. Arch. Otolaryngol. Head Neck Surg. 135, 296–302. 10.1001/archoto.2009.2 19289710

[B46] Stoikes N. F. Dutton J. M. (2005). The effect of endoscopic sinus surgery on symptoms of eustachian tube dysfunction. Am. J. Rhinol. 19, 199–202. 10.1177/194589240501900214 15921221

[B47] Van Zele T. Claeys S. Gevaert P. Van Maele G. Holtappels G. Van Cauwenberge P.. (2006). Differentiation of chronic sinus diseases by measurement of inflammatory mediators. Allergy 61, 1280–1289. 10.1111/j.1398-9995.2006.01225.x 17002703

[B48] Wagner Mackenzie B. Waite D. W. Hoggard M. Douglas R. G. Taylor M. W. Biswas K. (2017). Bacterial community collapse: a meta-analysis of the sinonasal microbiota in chronic rhinosinusitis. Environ. Microbiol. 19, 381–392. 10.1111/1462-2920.13632 27902866

[B49] Weiss S. Xu Z. Z. Peddada S. Amir A. Bittinger K. Gonzalez A.. (2017). Normalization and microbial differential abundance strategies depend upon data characteristics. Microbiome 5:27. 10.1186/s40168-017-0237-y 28253908 PMC5335496

[B50] Wolvers D. Antoine J. M. Myllyluoma E. Schrezenmeir J. Szajewska H. Rijkers G. T. (2010). Guidance for substantiating the evidence for beneficial effects of probiotics: prevention and management of infections by probiotics. J. Nutr. 140, 698S−712S. 10.3945/jn.109.113753 20107143

[B51] Wright E. D. Agrawal S. (2007). Impact of perioperative systemic steroids on surgical outcomes in patients with chronic rhinosinusitis with polyposis: evaluation with the novel Perioperative Sinus Endoscopy (POSE) scoring system. Laryngoscope 117(11 Pt 2 Suppl. 115), 1–28. 10.1097/MLG.0b013e31814842f8 18075447

[B52] Zhou J. C. Zhang X. W. (2019). Akkermansia muciniphila: a promising target for the therapy of metabolic syndrome and related diseases. Chin. J. Nat. Med. 17, 835–841. 10.1016/S1875-5364(19)30101-3 31831130

